# Development and psychometric evaluation of the AI emotional engagement scale

**DOI:** 10.3389/fpsyt.2026.1921527

**Published:** 2026-09-07

**Authors:** Jiasui Xu, Mengjie Mao, Yan Yao, Wen Li, Qunsen Dai

**Affiliations:** 1 School of Physical Education, Shaoxing University, Shaoxing, China; 2 School of Education and Psychology, Shaoxing University, Shaoxing, China; 3 College of Tourism and Service Management, Nankai University, Tianjin, China; 4 Hangzhoushi Fuyangqu Technical School, Hangzhou, China

**Keywords:** AI, emotional engagement, human-computer interaction, reliability and validity testing, scale development

## Abstract

**Background:**

With the increasing integration of artificial intelligence (AI) technologies into daily life, the underlying mechanisms of emotional interactions between users and AI have emerged as a critical research topic in the field of human-computer interaction (HCI). Existing studies have predominantly adopted traditional technology acceptance models or satisfaction scales, which lack targeted applicability in capturing unique features of AI interactions, such as parasocial relationships and emotional dependence. The present study aims to develop a self-report instrument, the AI Emotional Engagement Scale (AEES), and examine its psychometric properties for assessing users’ level of AI Emotional Engagement with AI systems.

**Methods:**

This study was conducted based on Norman’s three-level theory of emotional design. Study 1 (N = 24) extracted core information from interview data using qualitative analysis, and generated an initial item pool through multiple rounds of expert review. Study 2 (N = 103) conducted a pre-test of the initial items to examine indicators including item quality and relevance, optimized the item design, and developed the formal item set. Study 3 (N = 924) verified the factor structure of the AEES via exploratory factor analysis (EFA) and confirmatory factor analysis (CFA), conducted reliability and validity tests of the scale, and finalized the factor structure of the 28-item, three-dimensional scale.

**Results:**

Results of EFA indicated that AI Emotional Engagement consists of three dimensions: the visceral level, behavioral level, and reflective level, which was consistent with the theoretical framework, with a cumulative variance contribution rate of 68.32%. Results of CFA demonstrated that the three-factor model had a good fit (*χ²/df* = 3.765, CFI = 0.91, TLI = 0.90, RMSEA = 0.077, SRMR = 0.0496). The overall AEES and its three dimensions exhibited good internal consistency reliability, with reliability coefficients ranging from 0.90 to 0.97.

**Conclusion:**

The AEES has favorable psychometric properties, and can serve as a reliable instrument for researchers and practitioners to assess users’ AI Emotional Engagement with AI systems.

## 1 Introduction

In the current digital era, artificial intelligence (AI) technologies are integrating into all domains of human life at an unprecedented rate. With their application scenarios expanding from smart home devices and intelligent office assistants to healthcare, education, and entertainment, AI has exerted a profound and far-reaching impact on human society (1). AI has evolved from a specialized technical tool into a normalized interactive phenomenon widely used by the general public in daily life. Unlike traditional technical tools, AI systems, empowered by natural language processing, affective computing, and deep learning technologies, are equipped with anthropomorphic expression, contextual understanding, and autonomous responsiveness. The interaction mode with users has shifted from the traditional unidirectional human-tool operation to bidirectional interaction with interpersonal-like attributes (2). This shift has made the emotional bond between users and AI a core factor influencing user behavior, technology acceptance, and long-term usage intention, and has also sparked widespread academic attention to the underlying mechanisms of AI affective interaction (3, 4).

Existing studies on AI user behavior have mostly focused on technical performance optimization, function realization, and influencing factors of technology acceptance. A large body of these studies has adopted the Technology Acceptance Model (TAM) proposed by Davis (5) and its extended models, with a primary focus on users’ cognitive judgments, usage intention, and behavioral intention, while neglecting the complex emotional experiences and deep psychological bonds formed by users during their interactions with AI (6). Although some studies have begun to examine emotional responses, emotional dependence, and parasocial interaction phenomena in AI use, a unified and clear core structure of the relevant construct has not yet been formed, and there is a lack of standardized measurement instruments that have undergone rigorous psychometric validation (7, 8). Traditional user satisfaction scales and general emotional engagement scales were developed for non-AI contexts, making it difficult to capture core features unique to AI interaction, such as parasocial nature, emotional attachment, and value identification. This limitation leads to a lack of consistency and comparability in the conclusions of relevant empirical studies (9).

Moreover, the affective development of AI technology has also brought a series of new challenges for users’ mental health and psychological well-being. On one hand, AI systems with affective simulation capabilities can provide emotional support and companionship, potentially serving as a buffer against loneliness or social anxiety for vulnerable populations (10). On the other hand, users’ emotional dependence on AI may gradually extend from the functional level to the emotional level, which may further affect users’ autonomous decision-making ability, emotional independence, and real-world social relationships (11). Excessive emotional engagement with AI could blur the boundaries between human and machine relationships, increasing the risk of digital emotional addiction and reducing the motivation for genuine interpersonal interactions (12). Against this background, the development of the AI Emotional Engagement Scale (AEES), which combines both theoretical rigor and contextual adaptability, can not only provide a standardized instrument for empirical research on AI affective interaction, but also offer practical guidance for optimizing the user experience of AI products and provide a new research perspective for exploring the impact of AI use on users’ mental health. In particular, Norman’s (13) three-level theory of emotional design, which conceptualizes affective responses into visceral, behavioral, and reflective levels, provides a systematic and highly applicable framework for this endeavor, as it aligns well with the multi-layered nature of human-AI interaction, ranging from immediate sensory impressions to deep value-based emotional bonding. This framework also enables a clear operationalization of emotional engagement that can be adapted to the unique characteristics of AI, including its interactivity, adaptability, and parasocial qualities.

## 2 Literature review

### 2.1 Emotional engagement

The concept of emotional engagement originated in organizational behavior, laying the foundation for its subsequent cross-domain extension. Kahn (14) first proposed the concept of emotional engagement, defining it as a psychological state in which employees achieve emotional bonding and freely express their authentic emotions within their work roles, which established the theoretical framework for research on emotional engagement in workplace contexts. Building on studies related to job burnout, Schaufeli et al. (15) subsequently defined emotional engagement as a persistent and positive affective state consisting of three core dimensions: vigor, dedication, and absorption. Based on these three dimensions, they developed the globally applied Utrecht Work Engagement Scale (UWES), which has become one of the most widely used instruments for measuring emotional engagement in the field of organizational behavior. With the deepening of research, the study of emotional engagement has gradually expanded from the single field of organizational behavior to a hot interdisciplinary area covering educational psychology, consumer behavior, human-computer interaction, and other disciplines (16).

Educational psychology is a core domain for the further expansion of emotional engagement research. In educational psychology, emotional engagement is defined as students’ positive or negative affective reactions to the school, academic tasks, teachers, and peers (17), marking the conceptual transition from workplace contexts to learning settings. Centered on this definition, the academic community has carried out relevant studies focusing on measurement instruments and promotion strategies for emotional engagement in educational contexts (17). Accordingly, emotional engagement has been consolidated as a core construct capturing individual context interactions, providing empirical support for its extension to more non-workplace scenarios.

The field of consumer psychology has realized the commercial scenario extension and application boundary expansion of emotional engagement theory. In consumer behavior research, emotional engagement is inextricably linked to involvement theory. Grounded in this theory, Wirth et al. (18) defined emotional engagement as the degree of emotional bonding, emotional resonance, and psychological attention an individual forms toward a specific object. This conceptual definition retains the core connotation of emotional engagement while being optimized to fit consumer scenarios. Bagozzi et al.’s (19) research further verified that emotional engagement serves as a critical driver of consumers’ decision-making, brand attitudes, and purchase intention. Subsequent studies have explored research topics such as consumers’ irrational decision-making, brand loyalty or attachment, and parasocial interaction behaviors in the context of user interactions with brands or products (20, 21). This line of research has expanded the commercial application boundaries of emotional engagement theory, verified the feasibility of individuals forming emotional bonds with non-interpersonal, product-related objects, and laid an empirical foundation for the extension of the construct to technology product scenarios in human-computer interaction.

The field of human-computer interaction (HCI) is the core position for the application of emotional engagement theory in digital technology scenarios. In HCI research, emotional engagement places greater emphasis on the process of emotional bonding that users gradually establish during their interaction with machines, and its research focus has undergone three major shifts: from instrumental rationality centered on machine performance, to users’ technical identification and satisfaction with technology, and finally to user emotional arousal and cognitive involvement with emotional bonding at the core (22). Among relevant theoretical frameworks, Norman’s (13) three-level model of emotional design provides a systematic structure for understanding this research shift. The model divides affective responses in human-product interactions into three levels: the visceral level, behavioral level, and reflective level. The visceral level refers to sensory and immediate emotional responses triggered by the product, most prominently reflected in the systematic application of visual aesthetic design (23). The behavioral level focuses on functional perception and operational experience during use, manifested in users’ experience of functionality, usability, efficiency, and sense of control during the interaction process (24). The reflective level involves the deep impact of the product on users’ self-cognition, values, and long-term emotional bonding, embodied in the memory-based and personalized design of human-computer interaction (25). This model fully covers the process of emotional engagement in technology interactions, and establishes a clear dimensional framework for emotional engagement in digital product scenarios.

In summary, while existing theoretical and measurement research on emotional engagement has formed systematic accumulation across multiple domains, it has notable limitations in adaptability to the emerging AI interaction scenarios. To date, the research contexts of emotional engagement have expanded from organizational workplaces to education, consumption, and traditional human-computer interaction, with a rich body of measurement instruments developed for corresponding scenarios (3, 26). However, these instruments are still mainly developed for traditional scenarios (9). In recent years, although some scholars have paid attention to the process and contextual characteristics of emotional engagement and attempted to capture users’ emotional experiences in technology interactions through multi-dimensional measurement frameworks (27, 28), their dimensional design and item content have not covered the core features of large language model interactions, resulting in limited applicability in the context of AI technology (9). Especially against the background of the rapid development of large language models represented by ChatGPT, Claude, and Gemini, the affective interaction capabilities of AI have achieved a qualitative leap, and the cross-context validity and theoretical generalizability of traditional emotional engagement scales are facing new challenges. Research on emotional engagement in emerging technology scenarios is in urgent need of new measurement instruments to advance subsequent empirical research on AI emotional engagement.

### 2.2 AI emotional engagement

AI Emotional Engagement is a core emerging research construct in the field of human-AI affective interaction in the generative AI era. The full-scenario penetration of generative AI technology has driven the shift of human-computer interaction (HCI) paradigms from the traditional instrumental task-oriented approach to a deep emotional bonding-oriented one. Alongside this shift, this construct has become a cutting-edge core topic for interdisciplinary research spanning HCI, positive psychology, computer science, and other disciplines. With AI systems equipped with contextual understanding, long-term memory, autonomous decision-making, and affective simulation capabilities widely integrated into daily life, user-AI interactions have broken through the pure boundary of function execution, forming a new type of interactive relationship with significant social attributes and affective dimensions (29, 30). Recent research has further demonstrated that AI systems can, under certain conditions, establish interpersonal closeness comparable to human interaction, though the perception of interacting with an AI, rather than a human, significantly modulates this effect (31). Meanwhile, the emergence of AI companionship has been conceptualized through a triadic framework encompassing functional, social, and emotional dimensions, reflecting the increasingly complex nature of human-AI relationships.

Centered on this emerging phenomenon, some scholars have conducted preliminary explorations of its connotation from different perspectives. Some studies have defined it as the strength of emotional bond formed by users during their interaction with AI (7), while others have operationalized it as users’ emotional dependence on AI and continuous usage intention (32). However, to date, the academic community has not yet reached a unified, clear, and consensus-based definition, nor has it clarified the core dimensions and essential characteristics of this construct. Building on existing research foundations and the unique attributes of AI interaction, the present study defines AI Emotional Engagement as a psychological state characterized by concurrent cognitive involvement, affective projection, and behavioral attachment, which is formed by users during their interaction with AI systems and goes beyond the instrumental attributes of AI. This state is a composite form of psychological involvement and emotional bonding built upon authentic emotional experiences and a virtual interactive object.

Ambiguous conceptual boundaries and construct confounding are core limitations of current research on AI Emotional Engagement. In existing studies, AI Emotional Engagement is frequently confounded with multiple adjacent constructs in terms of connotation and operational definition, which directly hinders the horizontal comparison and systematic integration of relevant research findings (8). From the perspective of core theoretical connotation, AI Emotional Engagement has essential differences from four categories of adjacent constructs.

First, it has boundary differences from general emotional engagement in traditional domains. Classical research on emotional engagement mostly focuses on workplace, learning, and consumption contexts, with its core being individuals’ sustained psychological involvement and resource investment in specific real-world goals and concrete activities. The formation of such engagement relies on clear realistic value objectives and stable real objects (15). In contrast, the interactive object of AI Emotional Engagement is a virtual intelligent system with affective simulation capabilities, and its connotation breaks through the real object boundary and single instrumental core of traditional emotional engagement.

Second, it is essentially different from emotional attachment in intimate relationships. Classic attachment theory indicates that emotional attachment is a stable bidirectional emotional bond formed between an individual and a real attachment figure. The establishment of such a bond is contingent on the authentic emotional experiences, autonomous will, and mutual responsibility constraints of the interacting subjects (33). However, the empathic expression and affective feedback of AI essentially originate from algorithmic fitting rather than internal affective drive, and cannot achieve complete bidirectional affective resonance and responsibility assumption. There are core differences between the two constructs in terms of object authenticity and affective bidirectionality. Recent empirical work has further advanced this distinction: Yang and Oshio (10) developed the Experiences in Human-AI Relationships Scale (EHARS) grounded in attachment theory, while Cheng and Yu (34) validated the AI Attachment Scale (AIAS) with a three-factor structure comprising emotional support, separation distress, and secure base, demonstrating that anthropomorphism serves as the strongest predictor of AI attachment orientations. These developments suggest that while attachment frameworks can illuminate human-AI relationships, AI attachment remains distinct from human-to-human attachment in certain aspects due to the absence of reciprocal emotional agency.

Third, it has a clear distinction from parasocial interaction in traditional media contexts. The core of parasocial interaction is the unidirectional, imaginative affective projection formed by users toward media figures, with the core characteristics of non-reciprocity, non-responsiveness, and staticity (35). In contrast, AI Emotional Engagement relies on AI’s capabilities of real-time interaction, contextual understanding, and dynamic personalized feedback to form a bidirectional, dynamic, and responsive sustained interactive relationship, breaking through the core limitations of traditional parasocial interaction. However, this distinction is becoming increasingly nuanced. Recent research has documented that generative AI is reshaping parasocial relationships (PSR) in the digital age, with users forming one-sided emotional bonds that nevertheless exhibit growing markers of attachment and increased intentions to seek future AI companionship (36). Moreover, AI agents with high social presence can enhance consumer engagement through parasocial relationships. This evolving landscape suggests that while AI emotional engagement transcends traditional parasocial interaction in terms of interactivity and responsiveness, it shares with it a degree of emotional asymmetry, a complexity that warrants careful theoretical delineation.

Fourth, it has a clear demarcation from the constructs of technology acceptance and user satisfaction. The core of the Technology Acceptance Model (TAM) and user satisfaction assessment lies in users’ rational cognition and outcome evaluation of the functional utility and ease of use of technical tools, which essentially focuses on the instrumental attributes of technology (5). AI Emotional Engagement, by contrast, goes beyond the instrumental attributes of AI. It focuses on the deep emotional bond and psychological involvement of users during their interaction with AI, and represents a deeper and more comprehensive psychological construct than the former two.

Beyond these four distinctions, AI Emotional Engagement also relates to, but should not be conflated with, several other important constructs in the human-AI literature. Trust in AI, for instance, has been extensively studied as a cognitive and affective judgment about the reliability and benevolence of AI systems. While trust may serve as a precursor to emotional engagement, the latter entails a deeper, more sustained psychological investment that transcends mere confidence in system performance. Anthropomorphism, the attribution of human-like characteristics to AI, has been identified as a strong predictor of both trust and emotional bonding, yet it represents a perceptual tendency rather than an emotional state per se. Emotional dependence on AI has emerged as a growing concern in the literature, with recent studies suggesting that rumination may help explain how mind perception leads to emotional dependence, and that perceived emotional support, particularly reduced loneliness, freedom from judgment, and a sense of privacy, is among the strongest predictors of attachment-related behaviors. Emotional dependence may be conceptualized as a potential outcome or extreme manifestation of high emotional engagement, rather than its conceptual equivalent. These conceptual distinctions reinforce the need for a dedicated measure of AI emotional engagement that captures its unique multidimensional nature.

Existing research has formed phased academic consensus on the influencing factors and theoretical frameworks of AI Emotional Engagement. At the level of influencing factors, a basic consensus has been reached in the academic community: the formation and intensity change of AI Emotional Engagement are jointly shaped by two dimensions: system characteristics and user characteristics. System-related factors include AI technical and design features such as anthropomorphic design, interaction naturalness, and affective feedback quality. User-related factors involve individual difference variables such as technical beliefs, personality traits, cultural background, and affective needs. These two dimensions jointly shape the manifestation form and intensity level of users’ AI Emotional Engagement (37).

At the level of theoretical frameworks, existing studies mostly adopt general frameworks such as the traditional TAM and uses and gratifications theory. However, these frameworks mainly focus on the instrumental utility of technology, and may not fully cover the core features unique to AI interaction, such as parasocial attributes and dynamic emotional bonding (6, 38). Alternative frameworks such as the Computers Are Social Actors (CASA) paradigm and Media Equation Theory, while acknowledging that users treat computers as social actors, primarily explain the socialness of technology perception rather than providing a structured account of the multi-layered emotional experience that unfolds over time. Similarly, the AIDUA (Artificial Intelligence Device Use Acceptance) model, though specifically designed for AI acceptance, remains largely cognitive and adoption-focused, with limited capacity to capture post-adoption emotional bonding. In contrast, Norman’s (13) three-level theory of emotional design provides a systematic and adaptable core theoretical framework for research on AI Emotional Engagement. This theory divides users’ affective experiences in interaction with products into three core hierarchical levels that are progressive and mutually influential. The visceral level focuses on users’ intuitive affective responses and first impressions formed based on sensory stimuli; the behavioral level focuses on users’ functional experience, interaction efficiency, and sense of control during use; the reflective level focuses on users’ deep cognition, meaning construction, and long-term emotional bonding after interaction. This theory has a natural logical fit with the dual perspective of system and user, and can realize the full-chain mapping from AI system design characteristics to users’ AI Emotional Engagement experience. Moreover, the framework’s applicability to AI contexts has been increasingly recognized in recent scholarship. Peng (26) applied Norman’s framework to analyze human-AI companionship design, while other researchers have integrated the three-level model with insights from media studies and AI ethics to understand emotional design in AI-driven media. Existing studies have also verified that the affective expression, dialogue coherence, and personalized feedback capabilities of AI systems can significantly improve users’ affective trust and psychological involvement, further confirming the applicability of this theoretical framework to research on AI Emotional Engagement (7, 39).

Existing research has carried out multi-dimensional preliminary explorations on the measurement of constructs related to AI affective interaction. In existing empirical studies, some scholars have adopted general scales to conduct quantitative analysis of AI affective-related constructs, while others have developed single-dimensional measurement instruments for segmented AI scenarios, accumulating methodological foundations for standardized measurement in this field (8). Overall, however, the academic community has not yet developed a multi-dimensional standardized measurement instrument that matches the core connotation of AI Emotional Engagement and is supported by a systematic theoretical framework. Recent scale development efforts, such as the Human-AI Affective Bonding Inventory (HAABI) (40), the AI Attachment Scale (AIAS) (34), the Experiences in Human-AI Relationships Scale (EHARS) (10), and the Emotional Trust in Artificial Intelligence Scale (CEIA) (41), have advanced the measurement of related but distinct constructs, yet none specifically targets the multidimensional nature of emotional engagement as defined in the present study. This gap underscores the timeliness and necessity of developing the AEES.

## 3 The present study

The widespread adoption of generative AI across diverse scenarios has made the standardized measurement of AI Emotional Engagement a critical prerequisite for advancing related empirical research. As human-computer interaction (HCI) has gradually shifted from a traditional instrumental task-oriented paradigm to one increasingly centered on emotional bonding, AI Emotional Engagement has emerged as a potentially important indicator for evaluating the quality of human-AI affective interaction, predicting users’ long-term usage behavior, and assessing users’ digital emotional well-being (12, 42). With extensive attention from scholars across multiple disciplines including psychology, HCI, and computer science, the body of relevant research findings has grown rapidly (4). Scientific, standardized, and contextually adapted measurement instruments are a core foundation for conducting empirical research on AI Emotional Engagement.

Despite the growing scholarly interest, existing research has not yet established a systematic theoretical framework specifically adapted to AI interaction scenarios, which may hinder the development of a corresponding measurement instrument. While Norman’s (13) three-level theory of emotional design has been recognized for its potential applicability to human-AI affective interaction research, as it provides a structured account of affective responses ranging from immediate sensory impressions to deep value-based emotional bonding, and Ngo (37) has proposed that the influencing factors of AI Emotional Engagement can be decomposed into system characteristics and user differences, existing research has not yet systematically integrated these two perspectives or achieved contextual adaptation of the theory in light of the unique attributes of AI interaction. On one hand, most studies still rely on general frameworks such as the traditional Technology Acceptance Model (TAM) and user satisfaction theory, which may not fully capture the complex features unique to AI interaction, such as parasocial attributes, dynamic affective feedback, and long-term emotional bonding (6, 38). On the other hand, few studies have clearly elaborated the specific connotation and dimensional orientation of Norman’s three-level theory in the context of AI Emotional Engagement. This theoretical gap limits the extent to which the dimensional structure and item content of a scale designed for this construct can be systematically justified.

Furthermore, existing research lacks a dedicated, standardized scale for AI Emotional Engagement that has been developed following established psychometric protocols. At present, the academic measurement of AI Emotional Engagement remains underdeveloped in two respects. First, the problem of contextual mismatch is notable: most studies directly adopt mature scales from general domains such as work engagement and brand emotional engagement without conducting adaptive revisions targeting the affective characteristics of AI interaction. Consequently, these instruments may not accurately capture users’ specific level of AI Emotional Engagement. Second, among the few existing self-developed instruments, methodological rigor is often insufficient. Many studies have not systematically followed the classical scale development paradigm proposed by Churchill (43), and tend to lack key steps such as qualitative item generation based on real user experiences, multi-round expert review for content validity, independent pre-testing for item analysis, and large-sample validation of factor structure (8). These methodological gaps make it difficult to ascertain whether the dimensional structures identified in those studies are stable and replicable.

To address these limitations, the present study adopts Norman’s (13) three-level theory of emotional design as the core framework, a choice justified by its systematic coverage of the multi-layered nature of human-AI interaction and its adaptability to the dual system-user perspective, as discussed in the literature review. Drawing on the unique attributes of AI interaction, the study clarifies the core dimensions and operationalized connotation of the AEES. Through a rigorous and standardized psychometric development process, following the classical paradigm proposed by Churchill (43), the scale is developed and comprehensively validated. Specifically, this study comprises three phases. Study 1 employs semi-structured interviews and qualitative content analysis to generate an initial item pool grounded in both the theoretical framework and real user experiences, followed by expert review for content validity assessment. Study 2 conducts a pre-test with an independent small sample to examine item quality, discriminatory power, and internal consistency, refining the scale for formal administration. Study 3 utilizes a large-sample split-half design to explore the factor structure via exploratory factor analysis (EFA), confirm the dimensional structure via confirmatory factor analysis (CFA), and comprehensively test the reliability and construct validity of the AEES. Through this stepwise procedure, the study seeks to establish a psychometrically sound instrument that can facilitate future empirical investigations into the antecedents, consequences, and underlying mechanisms of AI Emotional Engagement.

## 4 Study 1: initial development of items for the AEES

Study 1 aimed to identify and classify the affective and behavioral characteristics of individuals during their interaction with AI, based on Norman’s (13) three-level theory of emotional design and combined with the dual system-user perspective of AI interaction. Through qualitative research and multiple rounds of expert review, this study provided support for the generation of initial items in the development of the AEES.

### 4.1 Method

This study followed the classical scale development paradigm proposed by Churchill (43). First, a semi-structured interview outline was developed through literature analysis. Semi-structured in-depth interviews were conducted to collect data on users’ real AI interaction experiences, and an initial item pool was generated through qualitative coding. The generated items were then revised through multiple rounds of expert review and content validity testing to develop the initial scale.

#### 4.1.1 Participants

Study participants were recruited offline on July 10, 2025, and members of the public were invited to participate in a survey on AI Emotional Engagement. In total, 30 respondents completed the survey. To ensure that participants had established an interactive relationship with AI, we adopted an inclusion criterion based on usage duration. Specifically, participants were asked an open-ended question: How long have you been using AI? Only participants who reported using AI for 3 months or longer were included in the final sample. After screening, the final sample consisted of 24 participants, of whom 62.5% were male and 37.5% were female, with a mean age of 32.72 years (SD = 5.62) (see **Table 1**).

**Table 1:** Socio-demographic characteristics of the study sample.

Socio-demographic characteristics	Study 1(*N*=24)	Study 2(*N*=103)	Study 3 (*N*=924)		
			Study 3-1(*n_1_*=463)	Study 3-2 (*n_2_*=461)	All
Male	15	48	217	227	444
Women	9	55	246	234	480
Mean age(SD)	32.72 (5.62)	29.7 (7.2)	31.11 (6.65)	30.84(6.53)	30.9 (6.6)
Education Level %					
High school or below	0	32	23	25	24
College diploma & Bachelor’s degree	83	47	52	52	52
Master’s degree	17	15	19	17	18
Doctorate	0	6	6	6	6

#### 4.1.2 Procedure

To improve the ecological validity and content representativeness of the scale, and to avoid items being limited to theoretical frameworks and disconnected from users’ real usage experiences, the semi-structured interview method was adopted to collect experiential descriptions from real AI users. Based on Norman’s three-level theory of emotional design, the interviews were structured around the visceral, behavioral, and reflective levels, and a semi-structured interview outline was developed incorporating the design logic of the dual system-user perspective (see **Table 2**).

**Table 2:** Semi-structured interview guide for user experience of AI products (excerpts).

Dimension	Interview item
Visceral Level (System Sensory Design - User’s Intuitive Experience)	When using an AI product, what design feature left the deepest first impression on you? What was your intuitive feeling about it?
	When using an AI product for the first time, which interface designs triggered an immediate sense of comfort or aversion in you?
	What distinctly different intuitive feelings do AI products with different design styles bring to you?
Behavioral Level (System Functional Interaction - User’s Behavioral Experience)	In daily use, which interactive features of AI make you willing to use it consistently and continuously?
	In multi-round conversations, which features make you willing to prolong the interaction and use the product in greater depth?
	When AI has a comprehension deviation, what fault-tolerant designs will make you willing to keep trying?
Reflective Level (System Emotional Design - User’s Psychological Experience)	After long-term use, have you developed a special emotional bond with this AI product? What are your feelings about it?
	During long-term use, have you ever had the profound feeling of "being understood and empathized with" from AI?
	When you are in a low mood or facing a difficult decision, will you turn to this AI product to talk things through and seek help?

Interviews were conducted in a one-on-one format, either online or offline, with each interview lasting 30 to 45 minutes. With the informed consent of the interviewees, the entire interview was audio-recorded, and then transcribed verbatim to form interview texts totaling approximately 80, 000 Chinese characters.

### 4.2 Data analysis

#### 4.2.1 Qualitative content analysis

The transcribed interview texts were analyzed using conventional content analysis (44), an approach suitable for deriving categories and codes directly from the raw data when existing theory is limited or when the goal is to describe a phenomenon in its natural context. Given that AI Emotional Engagement is an emerging construct with limited prior empirical investigation, this approach allowed for the identification of themes grounded in participants’ actual experiences while remaining attentive to the three-level theoretical framework.

The coding process proceeded in four phases. First, two researchers independently read all 24 interview transcripts multiple times to gain a holistic understanding of the content. Second, the researchers independently identified meaning units, segments of text corresponding to distinct experiences or perceptions of AI interaction, and assigned initial codes to each unit. Third, the two researchers met to compare their coding results; disagreements were resolved through discussion until consensus was reached. Fourth, the agreed-upon codes were grouped into preliminary categories based on semantic similarity, and these categories were then mapped onto the three theoretically derived dimensions (visceral, behavioral, and reflective) through an iterative process of constant comparison.

To enhance the trustworthiness of the analysis, several strategies were employed. The researchers maintained reflexive memos throughout the coding process to document their interpretive decisions. Peer debriefing was conducted with a third researcher not involved in the initial coding to review a random sample of 20% of the coded transcripts, yielding an inter-coder agreement rate of 86%, indicating satisfactory reliability. The resulting codes and categories were then used to transform users’ colloquial descriptions into standardized, measurable scale items. This process ensured that the items were closely aligned with real AI interaction scenarios.

#### 4.2.2 Item generation and expert review

Through literature review and coding of semi-structured interview texts, an original item pool containing 180 items was initially constructed. This included 52 items for the visceral level, 72 items for the behavioral level, and 56 items for the reflective level, with item content covering the three-level theoretical framework from the dual system-user perspective. Items for the visceral level covered the multimodal sensory design of AI systems and users’ intuitive affective responses; items for the behavioral level covered the interactive functional characteristics of AI systems and users’ behavioral involvement experiences; items for the reflective level covered the affective design of AI systems and users’ emotional bonding with AI.

To illustrate the transformation from raw interview data to scale items, two representative examples are provided. When a participant stated, *When I open the app, the clean interface and smooth animations immediately make me feel at ease*, this was transformed into the visceral-level item, *The visual design of the AI interface gives me a sense of comfort*. When another participant mentioned, *The AI remembers what I asked last time and follows up on it, which makes me feel like it really cares*, this was transformed into the reflective-level item, *The AI is able to follow up on topics from our previous conversations, which makes me feel understood*. These examples demonstrate how users’ colloquial expressions were standardized into item language while preserving the core meaning of their experiences.

After classifying the initial items by dimension, the 180 items were distributed to three independent experts for review. The three experts specialized in psychology, computer science, and human-computer interaction, respectively. During the review process, the content validity index was employed for quantitative assessment. After the first round of expert review, the overall scale-level content validity index of the original item pool was 0.82, which did not meet the desirable standard of 0.90. Specifically, 28 items were directly deleted because their item-level content validity index fell below 0.78; another 46 items were revised or rewritten based on the experts’ qualitative feedback due to ambiguous semantics, unclear dimensional attribution, or redundant wording; in addition, another 38 items were identified as having highly similar connotations. After the first round, the item pool was reduced to 152 items and proceeded to the second round of review.

In the second round, the experts conducted a further qualitative review of the 152 items retained after the first round. Based on their feedback, the 46 items that had been revised or rewritten in the first round were retained, as their content validity met the required standards. Meanwhile, the 38 items identified in the first round as having highly similar connotations were consolidated into 14 items, which were also retained because their content validity reached the required standards. At the same time, 22 items were removed for failing to be unequivocally mapped onto any of the three theoretical levels, indicating insufficient alignment with the core framework; 20 items were excluded because they predominantly reflected users’ instrumental evaluations of AI functionality or performance rather than their affective experiences, and were therefore considered conceptually closer to technology acceptance or satisfaction constructs and essentially distinct from the construct of emotional engagement; 18 items were eliminated because their wording was overly abstract and lacked contextual grounding in specific AI interaction scenarios, which might compromise respondents’ accurate understanding of the intended meaning; and the remaining 24 items were deleted due to content overlap with retained items or lower clarity compared to similar items. The remaining 8 items were unanimously judged by the experts to be clearly worded, well-grounded in theory, and closely tied to AI interaction contexts, and were therefore preserved without modification.

After two iterative rounds of review, the overall scale-level content validity index of the revised item pool improved from 0.82 to 0.93, reaching the desirable standard, and all retained items had item-level content validity indices of 0.78 or above, meeting the statistical criteria for item retention. Ultimately, the original 180-item pool was streamlined to 68 preliminary items, consisting of 46 items retained from the first-round revisions, 14 items generated through consolidation, and 8 items left unchanged. The dimensional distribution was 22 items for the visceral level, 28 for the behavioral level, and 18 for the reflective level. The scale adopted a 5-point Likert scoring format, with 1 = Strongly disagree, 2 = Somewhat disagree, 3 = Neutral, 4 = Somewhat agree, and 5 = Strongly agree. All items were positively scored. The items of the scale were concise and clear in expression, explicit in dimensional attribution, and close to real AI usage scenarios in content. With favorable content validity, the scale was suitable for entering the pre-test phase to conduct standardized item analysis.

## 5 Study 2: pre-test and item analysis of the AEES

Study 2 aimed to conduct standardized item analysis on the initial items of the AEES through an independent small-sample pre-test, to examine the discriminatory power, corrected item-total correlation, and internal consistency of the items. Poor-quality items were removed, and the scale structure and dimensional balance were optimized. This process ensured that the retained items aligned with the core logic of Norman’s three-level theory of emotional design and the dual system-user perspective, and formed the formal version of the AEES suitable for large-scale formal administration.

### 5.1 Method

#### 5.1.1 Participants

This study adopted a convenience sampling method to recruit respondents offline, with inclusion criteria consistent with those in Study 1. A total of 103 respondents with more than 3 months of continuous AI usage experience were recruited to participate in the pre-test. The sample size was considered appropriate for preliminary item analysis, as it exceeded the commonly recommended minimum of 100 respondents for pre-test item evaluation (45), while remaining manageable for the initial screening phase. All respondents signed an informed consent form before the administration, voluntarily participated in the study, and completed the questionnaire independently. The sample included 48 males and 55 females, with a mean age of 29.7 years (SD = 7.2) (see **Table 1**). The sample structure was broadly consistent with that of the formal administration sample in terms of age distribution and AI usage experience, which supported the applicability of the pre-test results for subsequent scale refinement.

#### 5.1.2 Procedure

The pre-test was conducted from July 25 to August 10, 2025, using a unified online questionnaire administration format. The questionnaire consisted of three parts, instructions, demographic information, and the pre-test scale composed of 68 items. The instructions clearly stated the research purpose, anonymity and confidentiality principles, response methods, and notes, and emphasized that there were no right or wrong answers to any items, and respondents should answer based on their real feelings. The average time for respondents to complete the questionnaire independently was 8 to 10 minutes.

### 5.2 Data analysis

#### 5.2.1 Descriptive statistics

Descriptive statistics of the pre-test data showed that the mean values of the 68 items ranged from 3.02 to 4.18, standard deviations ranged from 0.85 to 1.30, skewness coefficients ranged from -1.12 to 0.37, and kurtosis coefficients ranged from -0.82 to 1.15. The data distribution of all items met the requirements of approximate normal distribution, satisfying the statistical assumptions for item analysis.

#### 5.2.2 Item analysis

Item testing was conducted through corrected item-total correlation analysis and the extreme group method. The corrected item-total correlation coefficient reflects the extent to which each item is associated with the overall scale score; items with low correlations may not effectively measure the target construct. The extreme group method compares item scores between participants with the highest and lowest total scores; items that fail to differentiate between these groups lack sufficient discriminatory power.

The *a priori* item exclusion criteria were set as follows: (a) the correlation coefficient between the item and the total scale score was < 0.30 (45); and (b) the difference in item scores between the high-score group (upper 27%) and low-score group (lower 27%) did not reach the significance level of *p* < 0.05. The 27% rule for extreme group division follows established psychometric practice, which balances the need for sufficient group differentiation while retaining adequate sample sizes for statistical testing (46, 47).

Results of the corrected item-total correlation analysis showed that 17 items had a correlation coefficient with the total scale score lower than 0.30, indicating that these items had a relatively weak association with the core construct of the scale and may not effectively measure users’ level of AI Emotional Engagement. These items were deleted according to the *a priori* criteria. The extreme group method was used to test the discriminatory power of the items, and the results showed that the score difference of 12 items between the high-score group and low-score group was not statistically significant (*p* > 0.05), indicating that these items had insufficient discriminatory power to effectively distinguish respondents with different levels of AI Emotional Engagement. These items were also deleted according to the *a priori* criteria.

#### 5.2.3 Item screening and finalization of the formal scale

Building on the 29 items removed via quantitative statistical screening, further theoretical and structural optimization was conducted based on Norman’s three-level theory of emotional design and the dual system-user perspective, resulting in the removal of 11 additional items across three distinct categories. Specifically, 5 items were deleted due to highly overlapping semantic content and redundant measurement connotation within the same dimension, to avoid repeated measurement of the same construct and improve the conciseness of the scale. Three items with ambiguous dimensional attribution were excluded, these items could not be clearly mapped to a single theoretical level, which would have reduced the discriminability of the dimensional structure. The remaining 3 items showing potential cross-loadings in preliminary factor analysis were also removed, to eliminate items that might interfere with the stability of the factor structure in the subsequent formal large-sample test.

Taking all screening criteria together, a total of 40 items were deleted, and 28 items were finally retained to form the formal AEES (see **Table 3**). The formal scale included 10 items for the visceral level, 12 items for the behavioral level, and 6 items for the reflective level. The number of items was reasonably distributed across dimensions, consistent with the *a priori* three-dimensional theoretical framework. All retained items had a corrected item-total correlation coefficient ≥ 0.35, and all passed the discriminatory power test at the significance level of *p* < 0.001, suggesting that they can effectively measure users’ level of AI Emotional Engagement.

**Table 3:** Item content and dimensional structure of the AI emotional engagement scale (AEES).

Dimension	Item content	Theoretical rationale for classification
Visceral Level (V)	V1 Different AI systems vary in their service quality.	Belongs to the sensory stimulus to affective response pathway.
	V2 Other AI systems are equally intelligent.	A holistic cognitive judgment at the sensory level.
	V3 Using other AI systems would be equally enjoyable.	An immediate emotional response triggered by sensory attributes.
	V4 The font and icon designs of the AI are clear and easy to recognize.	Core focus on appearance and perception.
	V5 The layout of long text responses from the AI makes me feel at ease when reading.	Sensory design directly triggering affective response.
	V6 When I make an error, the AI’s text or voice prompts do not make me feel annoyed.	Sensory stimulus-triggered emotional response.
	V7 Necessary dynamic effects, such as loading animations, help alleviate my anxiety while waiting.	Sensory attributes directly modulating affect.
	V8 The color scheme of the AI interface gives me a pleasant feeling.	Direct measure of appearance and perception.
	V9 The visual feedback from the AI, such as animation effects, appeals to me.	Immediate affective response triggered by sensory stimuli.
	V10 I am more willing to use the AI because of its interface design.	Remains within the sensory stimulus to immediate response pathway.
Behavioral Level (B)	B1 The response speed of this AI satisfies me.	Core focus on functionality and usability.
	B2 This AI can accurately understand my vague expressions or instructions.	Functional usability evaluation at the behavioral level.
	B3 The personalized recommendations or suggestions from this AI meet my needs.	Measure of usage process and experience.
	B4 I am very likely to try new AI systems.	A behavioral-level openness focusing on behavioral intention.
	B5 I spend a long time using AI assistants.	Direct measure of usage behavior.
	B6 I invest a lot of effort in interacting with AI.	Behavioral measure of degree of investment during use.
	B7 I have invested a lot of resources in order to use AI.	Direct indicator of usage intensity.
	B8 The frequency of my daily interactions with AI is very high.	Direct measure of interaction quantity.
	B9 When I feel lost, I actively seek advice from AI.	Focuses on behavior occurrence rather than emotional bond depth.
	B10 When learning new things, I instinctively use AI to help me understand.	Focuses on the degree of behavioral automation in tool invocation.
	B11 When I feel bored, I open AI to chat and pass the time.	Focuses on behavior occurrence rather than emotional fulfillment.
	B12 When I encounter unexpected problems, I instinctively open AI first to seek help.	Focuses on behavioral priority rather than emotional dependence.
Reflective Level (R)	R1 The functions of this AI meet my usage needs well.	A cognitive evaluation of value identification.
	R2 The services provided by this AI are important to my work and daily life.	Captures the reflective process of value and meaning construction.
	R3 Using this AI has significantly improved my efficiency.	A post-use cognitive confirmation and meaning assignment of AI’s value.
	R4 The presence of AI makes me more confident in handling complex problems.	A value internalization process of AI meaning to self-concept.
	R5 Interacting with AI has given me a clearer understanding of my own needs.	Captures the reflective level’s facilitation of self-meaning construction.
	R6 I actively provide feedback and suggestions for the optimization of AI.	Captures deep psychological investment at the emotional maintenance level.

## 6 Study 3: formal administration and psychometric validation of the AEES

Based on the results of Study 2, Study 3 aimed to validate the factor structure of the AEES. Using a split-sample method, exploratory factor analysis (EFA) was conducted to explore the latent dimensional structure of the scale and verify its consistency with Norman’s three-level theory and the dual system-user perspective. Confirmatory factor analysis (CFA) was performed to examine the construct validity of the scale. Meanwhile, the internal consistency reliability of the scale was tested to verify the reliability and validity of the instrument.

### 6.1 Method

#### 6.1.1 Participants

This study adopted a convenience sampling method to recruit participants with more than 3 months of continuous AI usage experience for formal administration in AI-related enterprises, research institutions, and offline community settings. Convenience sampling was considered appropriate for this initial validation study, as the primary objective was to establish the factor structure and psychometric properties of the AEES in a sample with sufficient AI interaction experience, rather than to estimate population parameters or make broad generalizations. While this approach has recognized limitations in representativeness, it is commonly employed in the early stages of scale development to efficiently obtain data from the target population of active AI users (48).

A total of 1027 questionnaires were distributed, and strict data cleaning was conducted after collection. Invalid questionnaires with straight-lining responses, more than 10% missing items, and inconsistent response logic were excluded. Finally, 924 valid questionnaires were obtained, with an effective response rate of 89.97%. The valid sample included 444 males and 480 females, with a mean age of 30.9 years (SD = 6.6) and an age range of 18 to 58 years (see **Table 1**). All participants signed an informed consent form before administration, and were clearly informed of the research purpose, data usage, and voluntary participation principle, with the right to withdraw from the study at any time. The entire research process complied with the ethical guidelines for psychological research. To meet the independent sample requirement for EFA and CFA, this study used a random split-half method to divide the total sample into two independent subsets: Sample 1 (*n_1_* = 463) for exploratory factor analysis, and Sample 2 (*n_2_* = 461) for confirmatory factor analysis. This split-half design helps to avoid overfitting and enhances the replicability of the factor structure (49).

#### 6.1.2 Procedure

Formal administration was conducted from August 15 to August 28, 2025, with data collected through a combination of online and offline methods. The questionnaire consisted of three parts: the informed consent form, demographic information, and the AEES composed of 28 items, with simplified Chinese as the administration language. The questionnaire instructions were unified and standardized, which clearly stated the response requirements, anonymity and confidentiality principles, and research ethics. All items were scored on a 5-point Likert scale, with 1 = Strongly disagree and 5 = Strongly agree. The average time for participants to complete the questionnaire independently was approximately 10 minutes. After data collection, two researchers independently conducted data cleaning and coding to ensure data quality.

### 6.2 Data analysis

SPSS 27.0 statistical software was used for descriptive statistics, item analysis, exploratory factor analysis, and internal consistency reliability analysis. AMOS 24.0 statistical software was used for confirmatory factor analysis to examine the construct validity of the scale. Meanwhile, composite reliability (CR) and average variance extracted (AVE) were used to test the convergent validity of the scale, and the Fornell-Larcker criterion was applied to examine the discriminant validity of the scale. All statistical tests were two-tailed, with the significance level set at *p* < 0.05.

For model fit evaluation in CFA, five core indices were selected following the criteria proposed by Hu and Bentler (50), *χ²/df*, Comparative Fit Index (CFI), Tucker-Lewis Index (TLI), Root Mean Square Error of Approximation (RMSEA), and Standardized Root Mean Square Residual (SRMR). Good model fit was defined as *χ²/df* < 5, CFI ≥ 0.90, TLI ≥ 0.90, RMSEA < 0.08, and SRMR < 0.08. For internal consistency, Cronbach’s α coefficient ≥ 0.70 was considered acceptable, and ≥ 0.90 excellent (51). For convergent validity, CR ≥ 0.70 and AVE ≥ 0.50 were considered satisfactory (52).

### 6.3 Results and analysis

#### 6.3.1 Descriptive statistics and item analysis

Descriptive statistics of the 28 items in the formal AEES showed that the mean values of all items ranged from 3.46 to 4.10, standard deviations ranged from 0.93 to 1.24, skewness coefficients ranged from -1.07 to -0.48, and kurtosis coefficients ranged from -0.71 to 1.01. The data of all items met the requirement of approximate normal distribution, satisfying the assumptions for subsequent statistical analysis. Results of the extreme group method (upper 27% vs. lower 27%) showed that the score differences of all items between the high-score group and low-score group reached the significance level of *p* < 0.001, indicating that all items had adequate discriminatory power. Results of the corrected item-total correlation analysis showed that the correlation coefficients between each item and the total scale score ranged from 0.34 to 0.75, all reaching the significance level of *p* < 0.001, indicating that all items were sufficiently associated with the core construct of the scale and had satisfactory item quality (see **Table 4**).

**Table 4:** Means, standard deviations, skewness, kurtosis, and item-total correlations of the AI emotional engagement scale (*N* = 924).

Item	Mean	SD	Skewness	Kurtosis	t	r
B1	3.46	1.17	–0.49	–0.62	–23.82***	0.65***
B2	3.50	1.23	–0.62	–0.56	–28.66***	0.71***
B3	3.57	1.20	–0.64	–0.39	–28.25***	0.69***
R1	3.58	1.11	–0.63	–0.25	–9.85***	0.34***
R2	3.59	1.07	–0.48	–0.50	–12.08***	0.38***
R3	3.75	1.03	–0.62	–0.22	–12.60***	0.42***
V1	3.73	1.10	–0.49	–0.64	–13.86***	0.52***
V2	3.77	1.05	–0.77	0.23	–19.84***	0.59***
V3	3.87	1.06	–0.88	0.40	–14.28***	0.46***
B4	3.67	1.11	–0.74	–0.16	–25.77***	0.65***
V4	4.03	0.99	–1.07	1.01	–17.40***	0.54***
V5	3.85	1.00	–0.76	0.19	–16.36***	0.52***
V6	4.06	0.95	–0.94	0.65	–15.03***	0.45***
V7	3.82	1.03	–0.85	0.37	–14.80***	0.50***
V8	3.94	1.04	–0.84	0.21	–17.59***	0.55***
V9	3.95	0.99	–0.76	0.08	–13.91***	0.46***
V10	4.10	0.93	–0.89	0.42	–16.54***	0.55***
B5	3.55	1.23	–0.60	–0.61	–28.32***	0.69***
B6	3.71	1.13	–0.78	–0.11	–25.70***	0.69***
B7	3.51	1.21	–0.62	–0.57	–30.09***	0.75***
B8	3.62	1.16	–0.67	–0.34	–27.54***	0.71***
B9	3.60	1.24	–0.59	–0.71	–32.34***	0.75***
B10	3.54	1.20	–0.58	–0.62	–33.70***	0.72***
B11	3.65	1.14	–0.65	–0.41	–28.30***	0.71***
B12	3.73	1.20	–0.65	–0.67	–27.91***	0.72***
R4	3.63	1.06	–0.51	–0.26	–14.32***	0.48***
R5	3.78	1.06	–0.67	–0.15	–13.81***	0.42***
R6	3.69	1.10	–0.62	–0.23	–14.52***	0.46***

***p < 0.001; **p < 0.01; *p < 0.05.

#### 6.3.2 Exploratory factor analysis: exploration of dimensional structure

Data from Sample 1 were used for exploratory factor analysis to explore the number of latent factors, item attribution, and factor loadings of the scale, and to determine the dimensional structure adapted to the theoretical framework. First, the suitability test for factor analysis was conducted. Results showed that the Kaiser-Meyer-Olkin (KMO) value was 0.94, well above the critical standard of 0.7. According to the criteria proposed by Kaiser (53), a KMO value ≥ 0.9 indicates that the data are highly suitable for factor analysis. The result of Bartlett’s Test of Sphericity was significant (*χ²* = 12847.32, *df* = 378, *p* < 0.001), indicating that there were significant common factors among the items, which was suitable for factor analysis.

Subsequently, principal component analysis was used to extract common factors, and varimax orthogonal rotation was performed for factor rotation. An eigenvalue > 1 was set as the criterion for common factor extraction, and a factor loading ≥ 0.4 was set as the criterion for item retention. Results showed that 3 common factors with eigenvalues greater than 1 were extracted, which was consistent with the *a priori* three-dimensional theoretical framework of the visceral level, behavioral level, and reflective level. The cumulative variance contribution rate of the three factors was 68.32%, which could explain a substantial portion of the variance of the scale. The factor loading of all items on their corresponding factor was ≥ 0.75, well above the critical standard of 0.4. There were no items with cross-factor loadings, with clear item attribution and stable dimensional structure, which was largely consistent with the theoretical framework (see **Table 5**).

**Table 5:** Results of exploratory factor analysis of the AI emotional engagement scale.

Item	Factor 1	Factor 2	Factor 3	Communality
B1	0.07	**0.84**	0.04	0.71
B2	0.20	**0.85**	0.05	0.76
B3	0.15	**0.82**	0.08	0.71
R1	0.04	0.08	**0.82**	0.68
R2	0.17	0.15	**0.75**	0.62
R3	0.15	0.09	**0.82**	0.70
V1	**0.75**	0.11	0.13	0.59
V2	**0.77**	0.21	0.20	0.68
V3	**0.77**	0.10	0.06	0.61
B4	0.08	**0.86**	0.02	0.74
V4	**0.75**	0.15	0.12	0.59
V5	**0.81**	0.14	0.10	0.69
V6	**0.78**	0.06	0.14	0.64
V7	**0.77**	0.19	0.09	0.63
V8	**0.78**	0.12	0.10	0.63
V9	**0.76**	0.15	0.12	0.61
V10	**0.75**	0.25	0.16	0.65
B5	0.08	**0.84**	0.18	0.75
B6	0.17	**0.81**	0.13	0.70
B7	0.23	**0.83**	0.09	0.75
B8	0.09	**0.85**	0.13	0.75
B9	0.24	**0.85**	0.09	0.78
B10	0.19	**0.84**	0.16	0.77
B11	0.17	**0.82**	0.16	0.73
B12	0.14	**0.83**	0.12	0.72
R4	0.14	0.19	**0.78**	0.66
R5	0.19	0.08	**0.77**	0.64
R6	0.21	0.14	**0.78**	0.67
Initial Eigenvalues	4.73	11.39	3.02	–
% of Variance	16.88	40.66	10.78	–
KMO	0.94			–

Principal Component Analysis and Varimax Rotation (*n_1_* = 463). Bold values indicate the highest factor loading for each item and its primary factor assignment.

#### 6.3.3 Confirmatory factor analysis: test of construct validity

Based on the three-factor structure determined by EFA, data from Sample 2 were used for confirmatory factor analysis to examine the construct validity of the scale. To verify the superiority of the three-factor model, a one-factor model (all items loaded on a single common factor) was constructed as a competing model for model fit comparison.

Results of CFA showed that all fit indices of the three-factor model met the psychometric standards, with acceptable model fit: *χ²/df* = 3.765, CFI = 0.91, TLI = 0.90, RMSEA = 0.077, SRMR = 0.0496. In contrast, all fit indices of the competing one-factor model did not meet the standards: *χ²/df* = 14.745, CFI = 0.54, TLI = 0.51, RMSEA = 0.172, SRMR = 0.2088. The fit of the three-factor model was substantially better than that of the one-factor model, indicating that the three-dimensional structure of the scale had adequate construct validity (see **Table 6**).

**Table 6:** Model fit indices of the AI emotional engagement scale.

Model	*χ²*	df	*χ²*/df	CFI	TLI	RMSEA [90%CI]	SRMR
Model 1	5160.82	350	14.745	0.54	0.51	0.172 [0.168–0.177]	0.2088
Model 2	1306.53	347	3.765	0.91	0.90	0.077 [0.073–0.082]	0.0496

#### 6.3.4 Convergent validity and discriminant validity tests

Composite reliability (CR) and average variance extracted (AVE) were used to examine the convergent validity of the scale. According to the criteria proposed by Fornell and Larcker (52), satisfactory convergent validity is indicated by CR ≥ 0.70 and AVE ≥ 0.50. Results showed that the CR value of the overall scale was 0.96, and the CR values of the three dimensions (visceral level, behavioral level, reflective level) were 0.94, 0.97, and 0.91 respectively, all above the critical standard of 0.70. The AVE value of the overall scale was 0.58, and the AVE values of the three dimensions were 0.55, 0.61, and 0.52 respectively, all above the critical standard of 0.50, indicating that the scale had satisfactory convergent validity.

The Fornell-Larcker criterion was applied to test the discriminant validity of the scale, with the core criterion that the square root of the AVE value of each dimension should be greater than the correlation coefficient between that dimension and other dimensions. Results showed that the square root of AVE was 0.74 for the visceral level, 0.78 for the behavioral level, and 0.72 for the reflective level. The correlation coefficients between the three dimensions ranged from 0.42 to 0.67, all lower than the square root of the AVE of the corresponding dimension, indicating that the three dimensions of the scale had adequate discriminability and the scale had satisfactory discriminant validity.

#### 6.3.5 Reliability test

Internal consistency reliability (Cronbach’s α coefficient) was used to examine the reliability of the scale. According to the criteria proposed by Nunnally and Bernstein (51), a Cronbach’s α coefficient ≥ 0.70 indicates acceptable internal consistency, and a coefficient ≥ 0.90 indicates excellent internal consistency. Results showed that the Cronbach’s α coefficient of the overall scale was 0.94, and the Cronbach’s α coefficients of the three dimensions (visceral level, behavioral level, reflective level) were 0.93, 0.97, and 0.90 respectively, all above the excellent standard of 0.90, indicating that the scale had high internal consistency reliability, and the measurement results were stable and reliable.

## 7 Discussion

The present study developed and validated the AI Emotional Engagement Scale (AEES) following a rigorous psychometric procedure grounded in Norman’s (13) three-level theory of emotional design. Through a three-phase design, qualitative item generation, pre-test item analysis, and large-scale psychometric validation, the AEES demonstrated a stable three-factor structure corresponding to the visceral, behavioral, and reflective levels, with satisfactory internal consistency and construct validity.

### 7.1 Theoretical contributions and extensions

Based on Norman’s (13) three-level theory of emotional design, this study developed and validated the AEES from the dual perspectives of system perception and user experience, incorporating the unique parasocial characteristics and interactional complexity of AI technology to measure the emotional relationship between individuals and AI. Centered on Norman’s three-level theory of emotional design, the scale integrates elements such as perception of instrumental attributes and user affective experience that may emerge in AI contexts, and to a certain extent, addresses the theoretical gaps of traditional Technology Acceptance Model and general satisfaction scales in measuring AI user experience (6, 38). The results show that AI Emotional Engagement can be divided into a three-dimensional structure corresponding to the visceral, behavioral, and reflective levels, which align with users’ sensory experience, perception of functional interaction, and deep-seated value identification respectively. This finding is consistent with Ngo’s (37) argument that AI interaction has a high degree of social attributes.

From the perspective of theoretical integration, the development of the AEES facilitates the transfer of emotional engagement theory from traditional human-computer interaction contexts to AI contexts. Previous studies have often conceptualized emotional engagement as a unidirectional psychological response of users to technical systems. However, through the factor structure of the AEES, this study finds that users’ AI Emotional Engagement may stem from bidirectional interaction, which is not only influenced by system characteristics but also may depend on users’ cognitive traits and technology beliefs (30). This mechanism can partially explain how AI transcends the traditional role of a tool to establish a deeper emotional bond with users. Therefore, the findings of this study expand the application boundaries of emotional engagement theory in human-computer interaction scenarios, clarify the core dimensions of the emotional engagement construct in this context, and provide a standardized measurement instrument and a reference analytical framework for subsequent empirical research. Future studies may further explore the theoretical relationships between AI Emotional Engagement and other psychological variables.

The AEES also contributes to clarifying the conceptual boundaries between AI Emotional Engagement and related constructs. As discussed in the literature review, AI Emotional Engagement shares conceptual overlap with, but is distinct from, trust in AI, anthropomorphism, emotional dependence, and parasocial interaction. The empirical distinction observed in the factor structure, where visceral, behavioral, and reflective levels emerged as separable yet correlated dimensions, provides preliminary evidence that emotional engagement with AI is neither reducible to mere trust or satisfaction, nor synonymous with anthropomorphic perception. Rather, it appears to be a more encompassing psychological state that encompasses sensory, functional, and value-based layers of user-AI relating. This dimensional differentiation may offer a useful framework for future research seeking to disentangle the unique contributions of different aspects of the user-AI relationship to outcomes such as continued use intention, emotional well-being, or technology dependence.

### 7.2 Methodological rigor and psychometric value

The development of the AEES followed classical scale development procedures, combining qualitative and quantitative research methods to complete item generation, content validity assessment, pre-test, and large-sample empirical validation in a stepwise manner. Results from exploratory factor analysis (EFA) and confirmatory factor analysis (CFA) jointly supported the factor structure consisting of 3 dimensions and 28 items, with all fit indices (e.g., *χ²/df* = 3.765, CFI = 0.91, TLI = 0.90, RMSEA = 0.077) meeting psychometric standards, indicating that the scale exhibited adequate construct validity and stable factor structure in the study sample (54). In addition, Cronbach’s α coefficients for the overall scale and all dimensions exceeded 0.90, demonstrating high internal consistency reliability and providing strong support for the reliability of the AEES.

Compared with previous studies, this scale emphasized the integration of theoretical framework and real user experience during the item generation phase, and incorporated real user feedback through semi-structured interviews, which improved the content validity and ecological representativeness of the items. Meanwhile, the study used randomly split-half samples to conduct EFA and CFA respectively, which to a certain extent avoided the problem of overfitting and enhanced the replicability of the factor structure (49). This standardized scale development and validation process addresses part of the issues raised by Lintner (8) regarding the lack of systematic validation of AI affective measurement instruments, and provides a reference framework for subsequent scale development work in this field.

### 7.3 Practical implications and application prospects

The AEES has considerable application potential in scenarios of AI product design and user experience optimization. The three-dimensional structure of the scale can provide a reference framework for developers to deconstruct and evaluate users’ affective experiences at different levels, and may help developers identify shortcomings in user experience to achieve targeted product optimization. For example, at the visceral level, the visual appeal of interface design and interaction fluency may be key factors influencing users’ initial emotional responses (26), at the behavioral level, functional practicality and timeliness of feedback may directly affect users’ functional dependence and usage intention (55), at the reflective level, whether AI can trigger users’ emotional resonance and value identification may be a potential influencing factor of long-term user loyalty (7). Through dimensional assessment, enterprises can allocate resources more precisely and avoid neglecting the overall user experience due to excessive pursuit of technical performance.

In addition to product optimization, the scale may also be used to explore the potential impacts of AI technology on users’ mental health. For example, items related to emotional dependence and technology identification in the reflective level may help identify users with a tendency toward over-reliance on AI (11). In clinical or counseling contexts, the AEES could potentially serve as a screening tool to assess the extent to which individuals form emotional bonds with AI systems, information that may be relevant when considering the role of AI in users’ broader social and emotional lives. For instance, elevated scores on the reflective dimension might suggest a heightened vulnerability to substituting AI relationships for human contact, which could be of clinical interest, particularly among individuals with social anxiety or loneliness. However, the practical utility of the AEES in clinical settings remains to be empirically established through targeted validation studies. In educational AI applications, the scale may help instructors and designers understand how students affectively engage with AI tutors, potentially informing the design of systems that foster appropriate levels of engagement without encouraging overdependence.

Future studies may further examine the applicability and predictive validity of the scale in specialized scenarios such as education, healthcare, and psychological counseling, which may provide supporting reference for the rational deployment of AI systems and the assessment of users’ digital emotional well-being in these scenarios. Furthermore, the standardized three-dimensional structure of the scale provides a reference measurement instrument for subsequent cross-cultural and cross-population comparative studies on emotional engagement, and the scope of application of the scale can be further expanded through multi-group testing in the future.

### 7.4 Scoring and interpretation of the AEES

The confirmatory factor analysis in this study demonstrated that the three-factor model(CFI = 0.91, TLI = 0.90, RMSEA = 0.077, SRMR = 0.0496)fit substantially better than the one-factor model(CFI = 0.54, TLI = 0.51, RMSEA = 0.172, SRMR = 0.2088), with correlations among the three dimensions ranging from 0.42 to 0.67. This result indicates that the factor structure of the AEES exhibits a hierarchical relationship between global unity and multidimensional distinctiveness: the three dimensions collectively constitute the broad construct of AI emotional engagement, yet maintain theoretical distinguishability from one another. Accordingly, the interpretation of AEES scores requires differentiation between two levels: total scores and dimension scores.

The total score, calculated as the mean of all 28 items, reflects the overall level of users’ emotional engagement with AI systems. Although the one-factor model did not fit well, the moderate correlations among the three dimensions indicate that they share a certain amount of common variance, suggesting the presence of a measurable global construct. Therefore, when the research objective is to examine the overall intensity of users’ emotional engagement, such as comparing overall differences across user groups, or using emotional engagement as a predictor of distal outcomes, the total mean may be used. However, given the poor fit of the one-factor model, the total score should be interpreted as the average level of psychological investment across the three layers, rather than as a fully homogeneous unitary construct.

The dimension scores, calculated as the mean of items within each dimension, correspond respectively to three qualitatively distinct psychological processes. The visceral level mean reflects users’ immediate affective responses to AI sensory design, centering on the pathway between sensory stimuli and immediate responses, encompassing specific aspects such as interface aesthetics, interaction smoothness, and dynamic feedback. The behavioral level mean reflects users’ functional engagement and interaction investment during sustained use, centering on the process between operational behavior and interaction investment. The reflective level mean reflects users’ deeper value identification, meaning-making, and emotional bonding with AI systems, centering on the dimension of value internalization and meaning-making. The theoretical distinctiveness of the three dimension scores has been empirically supported by the CFA results: the three-factor model demonstrated good fit, and the square root of the AVE for each dimension exceeds its correlations with the other dimensions, indicating that each dimension score carries independent incremental explanatory value.

Based on the above analyses, the present study suggests the following: in exploratory research or when the overall effect of AI emotional engagement is of interest, researchers may report the total mean as a reference indicator of global level, while also reporting the three dimension means to present a more complete profile. In confirmatory or theory-driven research, particularly when hypotheses involve a specific psychological layer, such as the impact of interface design on sensory experience or the effect of value identification on usage stickiness, the corresponding dimension mean should be prioritized for hypothesis testing. These two types of scores address different research questions and should not be used interchangeably.

### 7.5 Research limitations and future directions

Although this study developed and validated the AEES through a rigorous procedure, there are still some research limitations that can be further refined in future studies.First, the sample of this study was recruited using the convenience sampling method, mainly from AI-related enterprises, institutions, and urban communities, and the sample was dominated by young and middle-aged, highly educated, and frequent AI users, resulting in certain sample selection bias. As noted by Rivera (56), convenience sampling has limited sample representativeness, which may affect the generalizability of the research findings. This demographic skew may also have influenced how participants perceived and responded to the visceral, behavioral, and reflective dimensions of emotional engagement. For example, younger and more technologically experienced users might be more attuned to interface aesthetics (visceral level) or more willing to attribute human-like qualities to AI (reflective level), potentially inflating the observed factor loadings or obscuring dimension-specific patterns that could emerge in more diverse samples. Future studies may further expand the sample scope to include elderly groups, groups with low technological literacy, and groups in rural areas, to improve the diversity and representativeness of the sample and examine the measurement invariance and applicability of the scale across different populations. Meanwhile, cross-cultural studies can be conducted to examine the applicability of the scale in different cultural contexts and promote the international application of the scale.

Second, this study only examined the construct validity, convergent validity, and discriminant validity of the scale, and did not test the criterion-related validity and test-retest reliability of the scale. Criterion-related validity would help demonstrate whether the AEES is actually associated with relevant external constructs such as established measures of emotional attachment, parasocial interaction, trust in AI, perceived social presence, or user-AI relationship quality. Test-retest reliability would provide evidence regarding the stability of the scale over time and whether emotional attachment to AI systems remains stable or fluctuates as users interact with the system over longer periods. Future studies may include criterion variables such as AI usage frequency, user stickiness, emotional attachment scales, and life satisfaction scales to examine the criterion-related validity of the scale and further verify its predictive power. Meanwhile, test-retest reliability can be examined through a longitudinal tracking design to verify the temporal stability of the scale’s measurement results.

Third, this study adopted a cross-sectional design, which can only examine the structure, reliability, and validity of the scale, but cannot reveal the dynamic process of users’ AI Emotional Engagement changes over time. Future studies may adopt a longitudinal tracking design or experimental intervention design to investigate the evolution trajectory of users’ emotional engagement at different stages of AI use, explore the influencing factors and long-term psychological effects of AI Emotional Engagement, and provide more in-depth empirical evidence for the theoretical development and specification formulation of AI affective interaction.

Finally, the pre-test phase in this study used a relatively small sample (N = 103). While this sample size exceeds the commonly recommended minimum of 100 for preliminary item evaluation (45), it may still be subject to sampling variability that could influence item-selection decisions. Some items deleted at this stage based on statistical criteria might potentially perform differently in larger or more diverse samples. Future studies may consider using larger pre-test samples or cross-validating the item selection process in independent samples to further ensure the stability and generalizability of the retained item set.

It should also be noted that the AEES was originally developed in Simplified Chinese, and all data collection and psychometric validation were conducted using the Chinese version. To facilitate international scholarly communication, the Chinese version was independently forward-translated into English by two bilingual psychology researchers, and then independently back-translated by two other researchers who had not participated in the forward-translation. The final English version was established through item-by-item comparison and discussion among all researchers until consensus was reached. The English items presented in **Table 3** are provided for reference purposes for international researchers. For applications in non-Chinese contexts, standardized translation and cross-cultural validation following established guidelines are strongly recommended.

## 8 Conclusion

This study completed the development and psychometric validation of the AEES, a self-report instrument for assessing users’ AI Emotional Engagement with AI systems. The results show that the scale has satisfactory psychometric properties, with a three-factor structure consistent with the visceral, behavioral, and reflective levels of emotional engagement. CFA supported this dimensional structure, and reliability analysis showed that the overall scale and all subscales had high internal consistency.

The AEES developed in this study demonstrates adequate psychometric properties as a self-report instrument for assessing the level of users’ AI Emotional Engagement with AI. The scale is easy to administer with a clear structure, making it suitable for academic research related to AI affective interaction. It also shows potential for application in fields such as AI user experience optimization and exploration of the psychological impacts of human-AI interaction relationships. Future research is needed to further establish its criterion-related validity, test-retest reliability, and cross-cultural applicability.

## Data Availability

The raw data supporting the conclusions of this article will be made available by the authors, without undue reservation.
